# The Association between Alexithymia and Social Media Addiction: Exploring the Role of Dysmorphic Symptoms, Symptoms Interference, and Self-Esteem, Controlling for Age and Gender

**DOI:** 10.3390/jpm13010152

**Published:** 2023-01-12

**Authors:** Alessio Gori, Eleonora Topino

**Affiliations:** 1 Department of Health Sciences, University of Florence, Via di San Salvi 12, Pad. 26, 50135 Firenze, Italy; 2 Integrated Psychodynamic Psychotherapy Institute (IPPI), Via Ricasoli 32, 50122 Florence, Italy; 3 Department of Human Sciences, LUMSA University of Rome, Via della Traspontina 21, 00193 Rome, Italy

**Keywords:** addiction, behavioral addiction, technological addiction, problematic social media use, body image, body dissatisfaction, body image concern, body dysmorphism, alexithymia, self-esteem

## Abstract

Given the popularity of social media and the growing presence of these tools in the daily lives of individuals, research about the elements that can be linked to their problematic use appears to be of great importance. The objective of this study was to investigate the factors that may contribute to the levels of social media addiction, by focusing on the role of alexithymia, body image concern, and self-esteem, controlled for age and gender. A sample of 437 social media users (32.5% men, 67.5% women; *M_age_* = 33.44 years, *SD* = 13.284) completed an online survey, including the Bergen Social Media Addiction Scale, Body Image Concern Inventory, Rosenberg Self-Esteem Scale, and Twenty-Item Toronto Alexithymia Scale, together with a demographic questionnaire. Results showed a significant association between alexithymia and social media addiction, with the total mediation of body image concern (and more in detail, body dissatisfaction) and the significant moderation of self-esteem. Gender and age showed significant effects in these relationships. Such findings may offer further insights into the field of clinical research on social media addiction and may provide useful information for effective clinical practice.

## 1. Introduction

In the last years, the use of social media has grown exponentially, becoming an integral part of everyday life for many individuals [1,2,3]. In fact, social media are online platforms that allow you to engage in many types of entertainment and social activities, for example, by socializing, creating content and interacting with that of others, and looking for information, without geographical or temporal constraints [4,5,6]. Although the scientific literature highlights a potential positive role of this medium on psychological well-being [7], attention has also been paid to some problematic outcomes that may affect some individuals, who may develop an addiction [8,9]. Social media addiction is a behavioral addiction linked to the use of technologies [10] defined as “*being overly concerned about social media, driven by an uncontrollable motivation to log on to or use social media, and devoting so much time and effort to social media that it impairs other important life areas*” (p. 4054) [11]. The symptoms of this condition include salience, tolerance, conflict, withdrawal, relapse, and mood modification [9,12], and this condition has been associated with higher levels of stress [13], depression [14], anxiety [15], sleep disorders [16], interpersonal problems [17], decreased work performance [18], and a lower life satisfaction [19].

Given the clinical relevance of this disease and its associated problems, the scientific literature has placed a growing focus on the exploration of risk and of the protective factors for this addiction (see Sun and Zhang [20] for a review). Studying the underlying elements of this pathological condition can provide valuable insights to identify the populations most at risk towards which to direct preventive interventions, on the one hand. On the other hand, it can support the efficacy of the clinical practice, expanding helpful knowledge to develop personalized therapeutic interventions on the characteristics of the subjects and targeted on the variables having a significant role in protecting or increasing the risk of resulting in problematic social media use. In this line, the present research examined the factors that may intervene in contributing to the levels of social media addiction, by focusing on the role of alexithymia, body image concern, and self-esteem.

Alexithymia is a multidimensional construct that falls within the framework of emotional dysregulation, and is characterized by a reduced ability to identify and describe feelings, difficulty in distinguishing feelings from bodily sensations, constrained imaginative processes, and poor introspective thinking [21,22]. It has been linked to both physical [23,24] and mental [25,26] illness, as well as the tendency to seek external regulation of one’s emotions through compulsive behaviors [21]. Consistently, scientific research has indeed highlighted its central role in contributing to vulnerability to the addiction onset [27] and its severity [28], whether it is substance abuse or problematic behavior [29,30]. More in detail, previous evidence also supports its association with technological addictions, including online gambling [31], problematic smartphone use [32], and, specifically, social media addiction [33].

Parallelly, the difficulty in identifying and symbolizing emotional experiences, as well as in distinguishing feelings from bodily sensations, can contribute to a perception of emotional emptiness, which in turn, may favor an exaggerated focus on the physical details of one’s body and an erroneous and distorted interpretation of one’s own body image [34,35]. In fact, previous evidence shows a significant and positive association between alexithymia and body image concern [36]. The body image construct refers to the perception and evaluation of individuals on their bodies [34]. On the other hand, dysmorphic concern is characterized by “*intense concern about, and preoccupation with, a perceived defect in appearance, excessive checking or camouflaging of the defect, social avoidance, and reassurance seeking*” [37] (p. 229). Previous evidence highlighted the negative influence of a negative perception of one’s body image on mental health, showing negative associations with depression [38], social anxiety [39], eating disorders [40], and exercise addiction [41], to name a few. Furthermore, recent research found relationships between body dissatisfaction and internet addiction [42], problematic smartphone use [43], and social media addiction [44], supporting the possibility that the use of the internet can represent a coping mechanism (maladaptive) aimed at temporary escape from unpleasant dysregulated feelings and negative perceptions of oneself, which in the long term may hesitate in addiction [45,46].

In this framework, it is not surprising that self-esteem has presented negative associations with social media addiction [19]. Self-esteem could be defined as a global evaluation of oneself that determines a positive or negative attitude towards the self [47]. It has been related to high mental health [48] and, in the field of addiction, it has negative relationships with substance use [49], gambling disorder [50], and exercise addiction [41], as well as technological addictions, such as internet addiction [51], internet gaming disorder [52], and problematic smartphone use [53]. In light of this evidence, the deepening of the protective role of self-esteem on social media addiction appears relevant, by exploring not only the direct associations between these two constructs but also the moderating effects of self-esteem in the relationships between other intervening factors. Investigating this aspect, in fact, could offer interesting insights of practical and clinical relevance.

Given the aforementioned evidence, this research investigated the relationships between factors that may influence the levels of social media addiction, focusing on alexithymia, body image concern, and self-esteem. To achieve this goal, a moderated mediation model was implemented, hypothesizing that:

**H_1_.**  *Alexithymia will be positively associated with social media addiction;*

**H_2_.**  *Body image concern will mediate the relationship between alexithymia and social media addiction;*

**H_3_.**  *Self-esteem will be a significant moderator in the model.*

Since previous studies have shown the influence of age and gender on the considered variables [54,55], these factors were controlled as covariates to test the solidity of the interactions hypothesized in the model.

Finally, the role of body image concern was deepened, by implementing a second model exploring the parallel mediation of the body image concern subdimensions (dysmorphic symptoms and symptom interference) in the relationship between alexithymia and social media addiction, further investigating the moderation of self-esteem, and controlling for the effects of gender and age.

## 2. Materials and Methods

### 2.1. Participants and Procedure

A sample of 437 social media users (32.5% men; 67.5% women) was involved in this research (see Table 1). Their mean age was 33.44 years (*SD* = 13.284), and most of them declared themselves to be single (59.5%), employees (36.4%), and have a high school diploma (36.6%). They were recruited online, by posting a link to the survey hosted on the Google Form platform. Participation was voluntary, and anonymity and privacy were guaranteed. Each participant was informed of the general objective of the research and provided electronically informed consent before starting the compilation. All the procedures of this research were approved by the Ethics Committee of the Integrated Psychodynamic Psychotherapy Institute (IPPI; protocol code 006/2022).

### 2.2. Measures

#### 2.2.1. Bergen Social Media Addiction Scale

The *Bergen Social Media Addiction Scale* (BSMAS) [56] is a six-item self-report measure used to assess the levels of problematic social network use, according to the conceptualization of the components model of behavioral addiction [12]. Items are rated on a five-point Likert scale, from 1 (“*very rarely*”) to 5 (“*very often*”). The Italian version [57] was used in this research, showing good internal consistency in the present sample (*α* = 0.80).

#### 2.2.2. Body Image Concern Inventory

The *Body Image Concern Inventory* (BICI) [37] is a 19-item self-report measure used to assess dysmorphic body image concerns. Items are rated on a five-point Likert scale, from 1 (“*never*”) to 5 (“*always*”), and may be grouped into two subdimensions: dysmorphic symptoms and symptom interference. Both the total score and the subscales of the Italian version [55] were used in this research, showing good internal consistency in the present sample (total score, *α* = 0.94; dysmorphic symptoms, *α* = 0.93; symptom interference *α* = 0.75).

#### 2.2.3. Rosenberg Self-Esteem Scale

The *Rosenberg Self-Esteem Scale* (RSES) [58] is a 10-item self-report measure used to assess the levels of personal self-esteem. Items are rated on a four-point Likert scale, from 0 (“*strongly disagree”*) to 3 (“*strongly agree*”). The Italian version [59] was used in this research, showing good internal consistency in the present sample (*α* = 0.89).

#### 2.2.4. Twenty-Item Toronto Alexithymia Scale

*The Twenty-Item Toronto Alexithymia Scale* (TAS-20) [60,61] is a 20-item self-report measure used to assess the levels of alexithymia. Items are rated on a five-point Likert scale, from 1 (“*strongly disagree”*) to 5 (“*strongly agree”*), and may be grouped into three subdimensions: difficulty identifying feelings; difficulty describing feelings; externally oriented thinking. The total score of the Italian version [62] was used in this research, showing good internal consistency in the present sample (*α* = 0.82).

**Table 1:** Demographic characteristics of the sample (*N* = 437).

Characteristics		M ± SD	N (*%*)
*Age*		33.44 ± 13.284	
*Sex*			
	Males		142 (32.5%)
	Females		295 (67.5%)
*Marital Status*			
	Single		260 (59.5%)
	Married		79 (18.1%)
	Cohabiting		81 (18.5%)
	Separated		3 (0.7%)
	Divorced		12 (2.7%)
	Widowed		2 (0.5%)
*Education*			
	Middle School diploma		17 (3.9%)
	High School diploma		160 (36.6%)
	University degree		115 (26.3%)
	Master’s degree		108 (24.7%)
	Post-lauream specialization		37 (8.5%)
*Occupation*			
	Student		103 (23.6%)
	Working student		59 (13.5%)
	Artisan		6 (1.4%)
	Employee		159 (36.4%)
	Entrepreneur		13 (3.0%)
	Freelance		39 (8.9%)
	Homemaker		11 (2.5%)
	Manager		5 (1.1%)
	Trader		2 (0.5%)
	Retired		22 (5.0%)
	Unemployed		18 (4.1%)

### 2.3. Data Analysis

Data were analyzed using the SPSS (v. 21.0; IBM, New York, NY, USA) software for Windows. Pearson correlation was used to examine the association between the variables. Then, two moderated mediation models were tested, by using the macro-program PROCESS 3.4 (Model 59) [63]. The first one was implemented to examine the relationship between alexithymia and social media addiction with the mediation of body image concern, also exploring the moderating role of self-esteem. The second model investigated the parallel mediation of the two body image concern subdimensions (dysmorphic symptoms and symptom interference) in the association between alexithymia and social media addiction, further exploring the moderating role of self-esteem. In both the models, it was controlled for gender (men coded as 0 and women coded as 1) and age as possible covariates. To probe the statistical stability of the models, the Johnson–Neyman procedure [64] and the bootstrap technique (5000 bootstrapped samples with 95% CI) [65] were used. The Johnson–Neyman procedure [64] allows the assessing of the conditional indirect effects of alexithymia on social media addiction at the three levels of self-esteem, i.e., −1 SD mean, +1 SD. The bootstrap technique provides bootstrapped confidence intervals (from boot LLCI to boot ULCI), which support the significance of the effect when they do not include zero [65]. Finally, the evaluation of the final moderated mediation model was enriched by examining the *R^2^* index, such that: *R^2^* < 0.02 = very weak effect; 0.02–0.12 = weak effect; 0.13–0.26 = moderate effect; *R^2^* > 0.26 = substantial effect [66].

## 3. Results

The correlation analysis showed significant associations among the variables (see Table 2). Specifically, social media addiction was significantly and positively correlated with alexithymia (*r* = 0.348, *p* < 0.01), body image concerns (*r* = 0.481, *p* < 0.01), dysmorphic symptoms (*r* = 0.474, *p* < 0.01), and symptom interference (*r* = 0.378, *p* < 0.01). Parallelly, social media addiction was significantly and negatively related to self-esteem (*r* = −0.369, *p* < 0.01).

A significant and positive total effect was found in the relationship between alexithymia and social media addiction (*β* = 0.33, *p* < 0.001; BootLLCI = 0.0978; BootULCI = 0.1702; **H_1_**), controlling for age (*β* = −0.32, *p* < 0.001; BootLLCI = −0.1419; BootULCI = −0.0845), and gender (*β* = 0.14, *p* < 0.001; BootLLCI = 0.6202; BootULCI = 2.2295).

As shown in the first moderated mediation model, alexithymia was also significantly and positively associated with body image concern (*β* = 0.44, *p* < 0.001; BootLLCI = 0.2852; BootULCI = 0.8585), which in turn, was significantly and positively related to social media addiction (*β* = 0.65, *p* < 0.001; BootLLCI = 0.0855; BootULCI = 0.3193; **H_2_**). Furthermore, self-esteem significantly moderated the association between body image concern and social media addiction (**H_3_**): ∆*R^2^* = 0.010, *F*(1, 429) = 6.290, *p* < 0.05. This data was further detailed by exploring the conditional indirect effects of alexithymia on social media addiction at three levels of the moderator (−1 SD, mean, +1 SD): the effect was significant at lower (estimate = 0.043; BootLLCI = 0.0218; BootULCI = 0.0681) or medium (estimate = 0.023; BootLLCI = 0.0107; BootULCI = 0.0392) levels of self-esteem, and became non-significant at higher levels of self-esteem (estimate = 0.009; BootLLCI = −0.0026; BootULCI = 0.0243). With regards to the covariates, being female was associated with higher levels of body image concerns (*β* = 0.22, *p* < 0.001; BootLLCI = 4.7926; BootULCI = 9.3005) and showed no significant relationship with social media addiction (*p* = 0.165; BootLLCI = −0.2390; BootULCI = 1.4025), unlike age, which was significantly and negatively related to both the variables (*β* = −0.16, *p* < 0.001, BootLLCI = −0.2514, BootULCI = −0.1028 and *β* = −0.26, *p* < 0.001, BootLLCI = −0.1188, BootULCI = −0.0620, respectively). Finally, when the mediation of body image concert with the moderation of self-esteem and the effect of gender and age were considered in the model, the direct effect in the relationship between alexithymia and social media addiction became non-significant (*β* = 0.30, *p* = 0.077; BootLLCI = −0.0160; BootULCI = 0.2673), suggesting for a fully moderated mediation explaining the 35% of the variance (a substantial effect): *R^2^* = 0.346, *F*(7, 429) = 22.417, *p* < 0.001.

**Figure 1:**
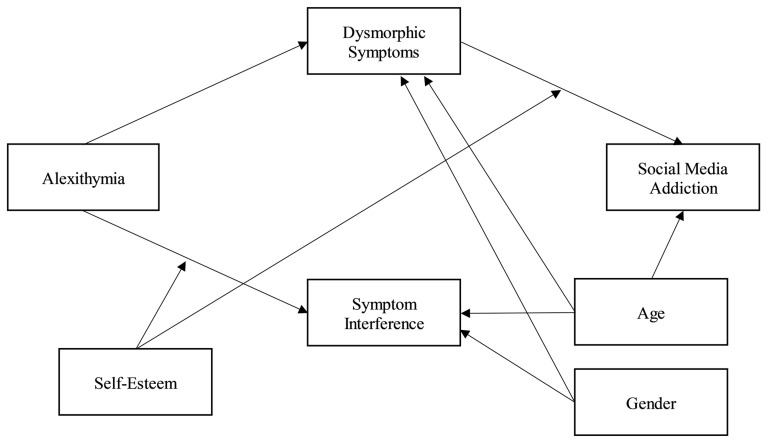
The association between alexithymia and social media addiction, exploring the role of dysmorphic symptoms, symptoms interference, and self-esteem, controlling for age and gender: a moderated mediation model. ***Note:*** Only the emerged significant relationships have been graphically represented.

**Figure 2:**
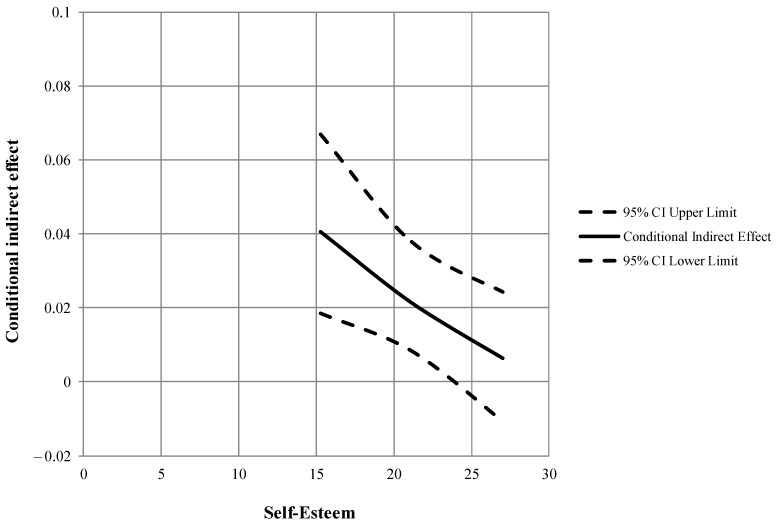
Conditional indirect effect of alexithymia on social media addiction at values of the moderator self-esteem through dysmorphic symptoms.

Concerning the results of the second moderated mediation model (see Figure 1), alexithymia was significantly and positively associated with the body image concerns subdimensions (dysmorphic symptoms, *β* = 0.35, *p* < 0.05; symptom interference, *β* = 0.75, *p* < 0.001). Furthermore, self-esteem significantly moderated the relationship between alexithymia and symptom interference, such that as self-esteem levels increased, the association between alexithymia and symptomatic interference weakened: ∆*R^2^* = 0.024, *F*(1, 431) = 15.149, *p* < 0.001. Then, dysmorphic symptoms were significantly and positively related to social media addiction (*β* = 0.76, *p* < 0.001), unlike symptom interference (*β* = −1.00, *p* = 0.596). In addition, the relationship between dysmorphic symptoms and social media addiction was significantly moderated by self-esteem: ∆*R^2^* = 0.010, *F*(1, 427) = 6.368, *p* < 0.05. This data was further detailed by exploring the conditional indirect effects of alexithymia on social media addiction at three levels of the moderator (−1 SD, mean, +1 SD; see Figure 2): the effect was significant at lower (estimate = 0.041; BootLLCI = 0.0185; BootULCI = 0.0669) or medium (estimate = 0.022; BootLLCI = 0.0083; BootULCI = 0.0378) levels of self-esteem, and became non-significant at higher levels of self-esteem (estimate = 0.006; BootLLCI = −0.0107; BootULCI = 0.0242).

With regards to the covariates, being female was associated with higher levels of dysmorphic symptoms (*β* = 0.23, *p* < 0.001) and symptom interference (*β* = 0.13, *p* < 0.01), and showed no significant relationship with social media addiction (*p* = 0.196), unlike age which was significantly and negatively related to both the body image concern subdimensions (dysmorphic symptoms, *β* = 0.17, *p* < 0.001; symptom interference, *β* = −0.09, *p* < 0.05) and social media addiction (*β* = −0.26, *p* < 0.001). Finally, when the mediation and moderation effects, as well as the influence of gender and age, were considered in the model, the direct effect in the relationship between alexithymia and social media addiction became non-significant (*β* = 0.34, *p* = 0.056), suggesting for a fully moderated mediation explaining the 35% of the variance (a substantial effect): *R^2^* = 0.348, *F*(9, 427) = 25.370, *p* < 0.001.( see Table 3)

## 4. Discussion

Given the rapid spread and popularity of social media [67], the scientific community is vigilant and active in investigating how the use of these tools can promote subjective well-being or, on the contrary, lead to problematic behaviors and disorders [68]. The present study fits into this line of research, focusing on the factors that may be associated with social media addiction by investigating the role of alexithymia, body image concern, and self-esteem, controlling for age and gender.

First, results showed a significant association between alexithymia and social media addiction, supporting the first hypothesis (**H_1_**). This is in line with previous evidence [69] and research on other technological addictions [70], further confirming the role of alexithymia as a risk factor for addiction [27,30] and, more generally, for psychopathology [71,72]. On the other hand, the data showed that the relationship between alexithymia and social media addiction was significantly and totally mediated by body image concern, according to the second hypothesis (**H_2_**). Indeed, alexithymic individuals tend to have reduced social skills, more difficulties in establishing and maintaining interpersonal relationships, lower perception of social support, and higher levels of anxiety in relational interactions [73,74], and can therefore perceive themselves as undesirable and physically inadequate [35]. From this perspective, social media can be seen as a safer and less risky context for developing relationships [75]. Indeed, the results of this research highlighted the significance of this indirect path only for subjects with lower levels of self-esteem, who played the role of moderator in the relationship between body image concern and social media addiction, in line with the third hypothesis (**H_3_**). This is consistent with previous evidence finding a significant mediation effect of self-esteem in the indirect path toward behavioral addiction [36,41] and showing that higher levels of self-esteem were associated with a lower perception of the importance of social media and a lower intensity of use [76]. Therefore, the data of this study suggest that individuals with lower self-esteem may rely on social media to compensate for their emotional and social deficiencies by seeking reassurance and socialization, risking more of developing an addiction to them [77].

Such findings were further investigated in the second model (see Figure 1), showing that the mediation of body image concern in the relationship between alexithymia and social media addiction was mainly explained by the significant effect of the dysmorphic symptoms subdimension, with the moderation of self-esteem. This data can be read considering that social media provides more control over self-presentation than face-to-face interactions [78], offering subjects with lower self-esteem the possibility to selectively share positive aspects of themselves to compensate for dissatisfaction with their own body [79]. In fact, previous evidence showed that subjects with higher body dissatisfaction tend to have a higher positive self-presentation on social media [80]. Therefore, social media becomes a compensatory solution to satisfy the psychological needs of individuals, limiting, however, the possibility of developing more adaptive strategies and increasing the levels of dependence on these tools [81].

Finally, the role of gender and age as potential confounders was controlled in performing both models. Concerning gender, it showed a significant effect on social media addiction when considered as a confounder of the total effect between alexithymia and social media addiction, in line with previous results, supporting that women present higher levels of problematic social media use than men [82]. However, when body image concern or its subdimensions were inserted in the models, being female was significantly associated with these factors and the direct effect on social media addiction became non-significant. These data provided further evidence of the greater risk of experiencing higher levels of body dysmorphism in women [55], and further detailed the scientific literature concerning the relationship between gender and social media addiction (e.g., [54]). With regards to age, older participants showed lower levels of both social media addiction and body image concern (or its subdimensions). Indeed, previous research highlighted that young people are more dedicated to using social media, increasing the risk of developing an addiction [83,84]. Furthermore, the relationship between body image concern and age was consistent with recent longitudinal research showing that satisfaction increased across the lifespan, both for men and women [85].

The present study also has some limitations that should be highlighted. First, the cross-sectional design of this study implies the need to be cautious in interpreting the causal links between the variables, however solidly based on the scientific literature. Longitudinal research is required to provide further evidence of these relationships. Furthermore, only self-report measures were used. The application of a multimethod approach (e.g., by integrating self-report measures with interviews) could be a strategy for future research to overcome the well-known method biases linked to self-reported data collection. Finally, a non-clinical sample was involved in this study. Although the conducted analyses allow for a dimensional approach, future research could replicate these models in clinical samples to confirm these results.

Despite these limitations, the results of this research may provide helpful insights for mental health professionals to elaborate tailored and personalized interventions. First, the association between social media addiction and body image concern has been further detailed, by considering the latter not only in its total dimension but also in its constitutive subdimensions [37]. This allowed highlighting the role of body dysmorphism, providing more specific information to favor a better understanding of the disorder. Furthermore, the results suggest the importance of evaluating the levels of alexithymia in subjects with social media addiction and the possibility of focusing on this aspect of the clinical intervention especially those who, precisely, also present body dysmorphism, confirming the importance of maintaining research attention on the study of alexithymia and emotional dysregulation in the field of addiction [27,30]. Finally, supporting the possibility of elaborating precise interventions both for clinical and preventive activities, the results also highlight a greater strength of the relationships between the risk factors for social media addiction in subjects with low self-esteem, in young people, and in women. Therefore, these data provide information on a specific target to which interventions should be directed, favoring their effectiveness and maximizing the available resources.

## 5. Conclusions

Although the use of the internet offers multiple opportunities and benefits, such as maintaining social relationships [4], remote working [86], and online therapy [87], to name a few, for some individuals this means also exposes them to the risk of developing technological addictions [88]. This study focused on social media addiction, highlighting the contribution of alexithymia, body image concern, and self-esteem through a moderated mediation model. The results offer interesting insights for clinical practice. Specifically, it should be noted that the relationship between alexithymia and social media addiction is totally mediated by body image concern (and, more specifically, by body dysmorphism), highlighting, however, that this path was significant only for subjects with lower self-esteem levels. This data has important practical implications for the planning of treatment interventions. In fact, although alexithymia and body image concern showed positive responses to therapy [89,90], the results of this study highlight the importance of working above all on self-esteem levels to limit the effect of these factors on social media addiction. Finally, the analysis of the confounders has made it possible to identify specific populations that may be most at risk, that is, women and younger subjects, towards which to direct any preventive activities. In conclusion, taken together these data offer further insights into the field of clinical research on social media addiction and may provide useful information for effective clinical practice.

## Figures and Tables

**Table 2:** Correlation Matrix.

	1	2	3	4	5	6
1. Social media addiction	1	0.384 **	0.481 **	0.474 **	0.378 **	−0.369 **
2. Alexithymia		1	0.471 **	0.457 **	0.408 **	−0.546 **
3. Body image concerns			1	0.990 **	0.773 **	−0.563 **
4. Dysmorphic symptoms				1	0.676 **	−0.547 **
5. Symptom interference					1	−0.485 **
6. Self-esteem						1

***Note***: Bold values indicate significant *p*-values. **. Correlation is significant at the 0.01 level (two-tailed).

**Table 3:** Unstandardized coefficients and 95% bias-corrected confidence interval of the totally moderated mediation model.

Antecedent	Consequent											
	M1				M2				Y			
	Coeff.	SE	*p*	95% BootCI	Coeff.	SE	*p*	95% BootCI	Coeff.	SE	*p*	95% BootCI
X	0.387	0.151	0.011	[0.1409; 0.6316]	0.185	0.036	<0.001	[0.1014; 0.2661]	0.137	0.071	0.053	[−0.0033; 0.2848]
W	−0.457	0.339	0.178	[−1.0178; 0.1050]	0.146	0.081	0.073	[−0.0339; 0.3207]	0.399	0.139	0.004	[0.1255; 0.6646]
X * W	−0.006	0.007	0.384	[−0.0175; 0.0051]	−0.007	0.002	<0.001	[−0.0100; −0.0029]	−0.003	0.003	0.406	[−0.0095; 0.0036]
M1	-	-	-	-	-	-	-	-	0.279	0.079	<0.001	[0.1090; 0.4503]
M2	-	-	-	-	-	-	-	-	−0.164	0.309	0.596	[−0.7710; 0.4180]
M1 * W	-	-	-	-	-	-	-	-	−0.009	0.004	0.012	[−0.0174; −0.0013]
M2 * W	-	-	-	-	-	-	-	-	0.010	0.016	0.052	[−0.0193; 0.0418]
C1	−0.160	0.037	<0.001	[−0.2256; −0.0933]	−0.018	0.009	0.040	[−0.0308; −0.0042]	−0.090	0.015	<0.001	[−0.1174; −0.0612]
C2	6.232	1.018	<0.001	[4.1992; 8.1766]	0.783	0.244	0.002	[0.2931; 1.2398]	0.536	0.414	0.196	[−0.2488; 1.3410]
Constant	290.494	80.134	<0.001	[16.2017; 43.3204]	−0.088	10.952	0.964	[−4.4608; 4.5435]	−10.083	30.339	0.746	[−7.2095; 5.1512]
	*R*^2^ = 0.418; *F*(5, 431) = 61.906, *p* < 0.001				*R*^2^ = 0.318; *F*(5, 431) = 40.252, *p* < 0.001				*R*^2^ = 0.348; *F*(9, 427) = 25.380, *p* < 0.001			

***Note:*** SE = standard error; Coeff = unstandardized coefficient; 95% BootCI = 95% bias-corrected confidence interval; X = alexithymia; M1 = dysmorphic symptoms; M2 = symptom interference; W = self-esteem; C1 = age; C2 = gender; Y = social media addiction.

## Data Availability

The data presented in this study are available on request from the corresponding author.
